# Efficacy of PD-1/PD-L1 plus CTLA-4 inhibitors in advanced/metastatic NSCLC: a meta-analysis based on RCTs

**DOI:** 10.3389/fimmu.2026.1833277

**Published:** 2026-05-11

**Authors:** Tianfu Dai, Yu Chen, Xuelian Dai, Yixin Chen, Xiaobo Pan, Jingqi Chen, Xingchen Liu

**Affiliations:** 1 The First Affiliated Hospital of Zhejiang Chinese Medical University (Zhejiang Provincial Hospital of Chinese Medicine), Hangzhou, Zhejiang, China; 2 The Graduate School, Zhejiang Chinese Medical University, Hangzhou, Zhejiang, China; 3 Xianju County Hospital of Traditional Chinese Medicine, Taizhou, Zhejiang, China; 4 Intensive Care Unit, Ningbo No.2 Hospital, Wenzhou Medical University, Ningbo, Zhejiang, China

**Keywords:** CTLA-4, meta-analysis, NSCLC, PD-1/PD-L1, prognosis, TMB

## Abstract

**Background:**

Dual immune checkpoint inhibitor (ICI) therapy (PD-1/PD-L1+CTLA-4 inhibitors) has emerged as a promising strategy, but its efficacy and optimal patient selection remain uncertain. This meta-analysis aimed to evaluate the efficacy of dual ICI therapy in advanced/metastatic non-small cell lung cancer (NSCLC) based on randomized controlled trials (RCTs).

**Methods:**

We systematically searched Cochrane Library, Embase, Web of Science, and PubMed from inception to August 2025 for RCTs comparing dual ICI therapy (PD-1/PD-L1+CTLA-4 inhibitors) versus control treatments in advanced/metastatic NSCLC. The primary outcome was overall survival (OS), and the secondary outcome was progression-free survival (PFS).

**Results:**

Ten RCTs comprising 6,369 patients were included. The OS (HR = 0.84, 95%CI 0.76-0.92, p=0.003) and PFS (HR = 0.78, 95%CI 0.68-0.89, p=0.002) of patients who received dual ICI treatment were significantly better than those in the control group. Subgroup analyses of the OS revealed significant benefits, including: squamous, non-squamous, PD-1 inhibitor, PD-L1 inhibitor, bone metastases, without bone metastases, male, age<65, ECOG = 0, Asian, PD-L1 TPS<1%, PD-L1 TPS≥50%, with high TMB (TMB≥10 or TMB≥20), smokers, brain metastases, without liver metastases, and when the control group received chemotherapy, placebo, first-line (1L) or non 1L (all p<0.05). Conversely, no significant benefit was observed in patients aged≥65, low TMB (TMB<10 or TMB<20), non-smokers, without brain metastases, or when the control group received monotherapy immunotherapy (all p>0.05 and P_interaction <0.05).

**Conclusion:**

Dual ICI therapy with PD-1/PD-L1 plus CTLA-4 inhibitors improves prognosis in advanced/metastatic NSCLC, with efficacy difference across subgroups. TMB may serve as a complementary predictive biomarker for dual immunotherapy.

**Systematic review registration:**

PROSPERO, identifier: CRD420251147483.

## Introduction

Lung cancer is the leading cause of cancer incidence and mortality. It accounts for nearly one-eighth (12.4%) of all cancer diagnoses worldwide and one-fifth (18.7%) of cancer-related deaths (1). Non-small cell lung cancer (NSCLC) constitutes approximately 89.5% of all lung cancer cases, encompassing squamous and non-squamous histological subtypes (2). Although therapeutic advances have gradually improved patient outcomes, the 5-year relative survival rate of patients with advanced NSCLC is only 6.1% (3). Current standard treatments for advanced disease include chemotherapy, radiotherapy, immunotherapy and targeted therapy. In recent years, immunotherapy—particularly inhibitors targeting the programmed cell death protein 1 (PD-1) and its ligand (PD-L1)—has markedly improved prognosis in NSCLC, raising the 5-year survival rate for advanced/metastatic disease to 13.4% (4).

Despite the significant reduction in mortality associated with PD-1/PD-L1 inhibitors, the prognosis for patients with advanced/metastatic NSCLC remains unsatisfactory. This has prompted the exploration of more effective treatment strategies, including combination regimens such as dual immune checkpoint inhibitor (ICI) therapy, dual targeted therapy, and immunotherapy combined with targeted agents. Previous studies have revealed that the PD-1/PD-L1 pathway mediates adaptive immune resistance in the tumor microenvironment (TME) (5). PD-L1 inhibits T-cell receptor signal transduction by recruiting SHP1/SHP2 protease inhibitors through binding to PD-1 on T cells, leading to T-cell exhaustion (6). Unlike the PD-1/PD-L1 pathway, CTLA-4 mainly regulates the early activation of T cells in lymphoid organs and functions by competitively inhibiting CD28 co-stimulatory signals and regulating regulatory T cell functions (7). Based on these mechanisms, CTLA-4 inhibitors can block the CTLA-4 pathway to expand the T-cell receptor repertoire (8), while PD-1/PD-L1 inhibitors can activate T cells in the TME by blocking the PD-1/PD-L1 pathway. The synergistic action of these two mechanisms may enables the dual ICI therapy to achieve good efficacy in patients with advanced/metastatic NSCLC.

In the context of dual ICI targeting PD-1/PD-L1 and cytotoxic T-lymphocyte-associated protein 4 (CTLA-4) in advanced/metastatic NSCLC, pivotal phase III trials such as CheckMate 227 (9) and CheckMate 9LA have demonstrated promising long-term survival benefits with regimens combining PD-1/PD-L1 and CTLA-4 inhibitors, either with or without a short course of chemotherapy. However, the recently published GFPC 08–2015 ENERGY (10) trial did not show a significant benefit when use nivolumab plus ipilimumab versus carboplatin-based doublet as first-line treatment in advanced/metastatic NSCLC.

Several recent meta-analyses have addressed overlapping questions in this field. Di Federico et al. (2025) (11), through an individual patient data meta-analysis of 6 trials published in *Lancet Oncology*, compared dual immune checkpoint inhibitors (ICIs) with ICI monotherapy and performed molecular subgroup analyses with rigorous methodology. Alifu et al. (2023) (12) and Zhao et al. (2025) (13) also evaluated dual ICI–based regimens in advanced NSCLC. Unlike these previous analyses, our study pools 10 randomized controlled trials (6,369 patients) comparing dual ICI ± chemotherapy versus non-dual-ICI controls, thereby capturing a broader and more clinically relevant question that reflects real-world therapeutic decision-making. Importantly, our analysis incorporates recently published trials—including JCOG2007 (Shiraishi et al., 2024) (14) and ENERGY (Léna et al., 2025) (10)—that were not available at the time of prior meta-analyses, providing updated evidence particularly relevant to elderly and East Asian populations. In addition, we conducted comprehensive subgroup and interaction analyses across 33 clinical and biomarker-defined strata, reporting gold-standard heterogeneity metrics including τ² with 95% confidence intervals (95% CI), I² with 95% CI, Cochran’s Q with P-values, and prediction intervals (PI), thereby offering a more granular characterization of treatment effect modifiers than previously available.

Therefore, to provide more comprehensive and updated evidence, this meta-analysis will systematically evaluate the efficacy of dual ICI therapy (PD-1/PD-L1+CTLA-4 inhibitors) in patients with advanced/metastatic NSCLC based on high-quality randomized controlled trials (RCTs).

## Methods

### Study registration

The present systematic review and meta-analysis were conducted in accordance with the Preferred Reporting Items for Systematic Reviews and Meta-Analyses (PRISMA) guidelines. The study protocol was registered with the International Prospective Register of Systematic Reviews (PROSPERO) under registration number CRD420251147483.

### Data sources and search strategy

From database inception to August 2025, a comprehensive literature search was conducted across four electronic databases: the Cochrane Library, Embase, Web of Science, and PubMed, to identify studies on PD-1/PD-L1 combined with CTLA-4 dual immunotherapy in patients with advanced/metastatic NSCLC. The search terms were combined medical subject headings (MeSH) and free words: ((advanced) OR (metastasis)) AND ((non-small cell lung cancer) OR (NSCLC)) AND ((Cytotoxic T-Lymphocyte-Associated Protein 4) OR (CTLA-4) OR (ipilimumab)) OR (tremelimumab)) OR (botensilimab)) OR (ADG126)) AND ((PD-1) OR (PD-L1) OR (programmed death 1) OR (programmed death ligand 1) OR (nivolumab) OR (BMS 936558) OR (BMS 936559) OR (MDX 1105) OR (pembrolizumab) OR (lambrolizumab) OR (MK 3475) OR (pidilizumab) OR (CT 011) OR (durvalumab) OR (MEDI 4736) OR (atezolizumab) OR (MPDL 3280a) OR (avelumab) OR (AMP 224) OR (toripalimab) OR (camrelizumab) OR (SHR-1210) OR (sintilimab) OR (tislelizumab) OR (penpulimab) OR (zimberelimab) OR (envafolimab) OR (cemiplimab)). To minimize the risk of omitting eligible publications, a manual search of the reference lists of included articles and clinical trials.gov was also performed. Two investigators independently conducted the literature search, and any discrepancies were resolved through consensus discussion.

### Inclusion criteria

Studies were selected based on the PICOS framework. The inclusion criteria were as follows: (1) patients suffered from advanced/metastatic NSCLC; (2) the experimental group received the intervention of PD-1/PD-L1 combined CTLA-4, with or without chemotherapy; (3) the control group was treated with other treatments including single immunotherapy, targeted therapy, chemotherapy, placebo or combination of them; (4) the results of the study was overall survival (OS) and progression-free survival (PFS); (5) study design belonged to RCT.

### Exclusion criteria

Studies that met any of the following characteristics were excluded: (1) if multiple publications reported on the same trial population, only the most recent or comprehensive version was included; (2) data was not available or cannot be subjected to statistical analysis; (3) articles not published in English; (4) full-text was unavailable.

### Data extraction

Data extraction was performed independently by two investigators using a pre-designed data collection form. Any discrepancies between reviewers were resolved through consensus discussion. The following information was extracted from each included study: authors, year of publication, inclusion criteria, sample size, treatment regimens, primary outcomes (including OS), secondary outcome (including PFS), and clinical trial registration number.

### Assessment of risk of bias

The risk of bias was assessed using the revised Cochrane Risk of Bias tool (RoB 2). Seven domains were evaluated: (1) random sequence generation (selection bias), (2) allocation concealment (selection bias), (3) blinding of participants and personnel (performance bias), (4) blinding of outcome assessors (detection bias), (5) incomplete outcome data (attrition bias), (6) selective reporting (reporting bias), and (7) other potential sources of bias. Each domain was judged as ‘low risk’, ‘high risk’, or ‘unclear risk’. Any disagreements between reviewers were resolved through consensus.

### Statistical analysis

Statistical analyses were performed using Stata 12.0 (StataCorp, College Station, TX, USA) and R 4.5 (R Core Team, Vienna, Austria). Hazard ratios (HR) with 95%CI were used to evaluate the relationship between the intervention plan and time-to-event outcomes. An HR≥1 indicated a benefit in favor of the control group, whereas an HR<1 suggested a benefit favoring the experimental group. Subgroup analyses were conducted based on prespecified factors, including sex, age, Eastern Cooperative Oncology Group (ECOG) performance status, pathological subtype, geographic region, PD-L1 tumor proportion score (TPS), smoking history, tumor mutational burden (TMB), immunosuppressant type, control group regimen, metastatic pattern and treatment line. Given the differences in study design, population baseline characteristics, and methodology among the studies, we adopted the Restricted Maximum Likelihood for estimating heterogeneity variance τ², and the Hartung-Knapp-Sidik-Jonkman method for confidence intervals. The heterogeneity was evaluated by Cochran’s Q test (χ2), the heterogeneity was quantified by the I2 statistic (I2≥50% indicates high heterogeneity; I2<50% indicates low heterogeneity), and the variance between studies (τ2) was further estimated to reflect the absolute degree of true effect changes across studies. In addition, the 95% prediction interval of the combined effect size was provided. Publication bias was assessed using Egger’s test, and sensitivity analysis was performed to evaluate the robustness of the pooled results. A two-sided p-value<0.05 was considered statistically significant.

## Results

### Study selection and characteristics

**Figure 1:**
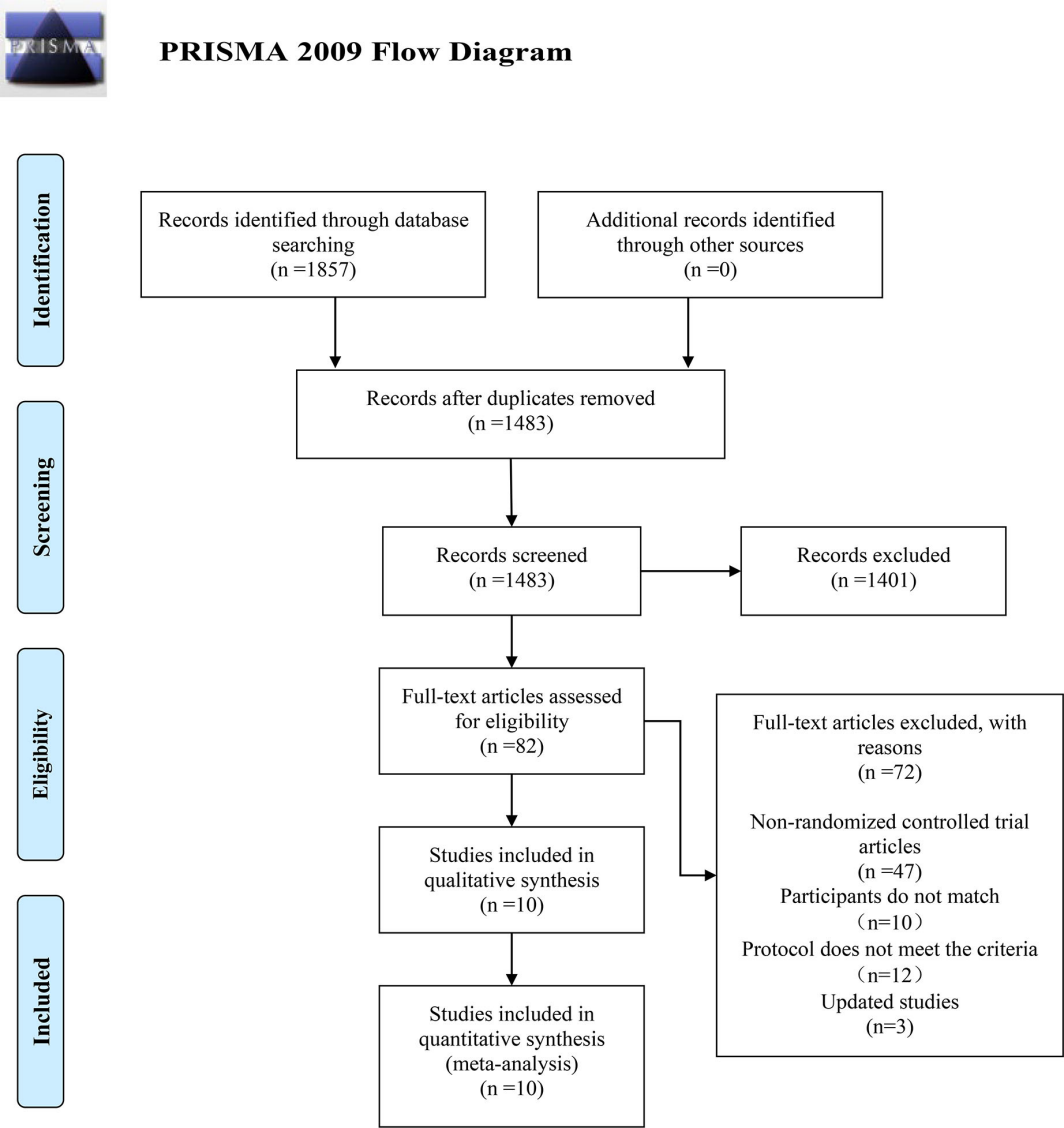
Flow chart of study selection.

The initial search across Cochrane Library, Embase, Web of Science, and PubMed yielded 1,857 records. No additional records were identified from the reference lists of included studies. After removing duplicates, 1,483 studies were screened based on their titles and abstracts. This led to the exclusion of 1,401 studies, resulting in 82 articles for full-text review. Of these, 72 were excluded for the following reasons: not being RCTs (n=47), having non-eligible study populations (n=10), employing non-eligible interventions or protocols (n=12), or being superseded by updated publications (n=3). Consequently, 10 studies were included in the qualitative synthesis and meta-analysis (10, 14–22). See the following **Figure 1** for details.

The 10 RCTs included, published in 2019-2025, included 6,369 samples. Among the included studies, the experimental groups in five received the nivolumab-ipilimumab (N-I) intervention, while four studies administered the durvalumab-tremelimumab (D-T) intervention to their experimental groups, and one study applied the pembrolizumab-ipilimumab (P-I) protocol. In the control groups, two studies involved with pembrolizumab, one study involved with nivolumab, two studies involved with durvalumab, and one study involved with either durvalumab or tremelimumab. The characteristics of the studies and treatment regimens are described in **Table 1**. Eight of the ten studies used dual ICI as first-line (1L) therapy, one as 2L therapy, and one as 3+L therapy. Patients had TNM stage III-IV disease. The tumor staging is based on both the American Joint Committee on Cancer (AJCC) and International Association for the Study of Lung Cancer (IASLC) standards. See **Supplementary Table 1** for further details.

**Table 1:** Characteristics of all the studies included in the meta-analysis.

Author	Year	RCT	Arm	Number of patients	Treatment regimens	PD-1/PD-L1 dosage regimen	CTLA-4 dosage regimen
Boyer M (15)	2021	KEYNOTE-598	experiment	284	pembrolizumab + ipilimumab	200 mg q3w up for to 35 doses	1 mg/kg q6w for up to 18 doses
			control	284	pembrolizumab + placebo		
Carbone D P (22)	2025	CheckMate 9LA	experiment	361	nivolumab + ipilimumab + chemotherapy	360 mg q3w	1 mg/kg q6w
			control	358	chemotherapy		
Gettinger S N (16)	2021	Lung-MAP(S1400)	experiment	125	nivolumab + ipilimumab	3 mg/kg q2w	1 mg/kg on day 1 of every third cycle
			control	127	nivolumab		
Léna H (10)	2025	GFPC 08–2015 ENERGY	experiment	109	nivolumab + ipilimumab	240 mg q2w	1 mg/kg q6w
			control	107	chemotherapy		
Hellmann M D (17)	2019	CheckMate 227	experiment	396	nivolumab + ipilimumab	3 mg/kg q2w	1 mg/kg q6w
			control	397	chemotherapy		
Cheng Y (18)	2023	NEPTUNE China chort	experiment	474	durvalumab + tremelimumab	20 mg/kg q4w	1 mg/kg q4w for up to 4 cycles
			control	479	chemotherapy		
Rizvi N A (19)	2020	MYSTIC	arm1	371	durvalumab + tremelimumab	20 mg/kg q4w	1 mg/kg q4w for up to 4 doses
			arm2	352	chemotherapy		
			arm3	369	durvalumab		
Johnson M L (20)	2023	POSEIDON	arm1	338	durvalumab + tremelimumab + chemotherapy	q3w for 12 weeks	q3w for 12 weeks
			arm2	338	durvalumab + chemotherapy		
			arm3	337	chemotherapy		
Shiraishi Y (14)	2024	JCOG2007	experiment	147	nivolumab + ipilimumab + chemotherapy	360 mg(N)/200mg(P) q3w	1 mg/kg q6w
			control	148	pembrolizumab + chemotherapy		
Planchard D (21)	2020	ARCTIC study B	arm1	173	durvalumab + tremelimumab	20 mg/kg q4w up to 12 weeks +10 mg/kg q2w for 34 weeks	1 mg/kg q4w for up to 12 weeks
			arm2	118	chemotherapy	/	/
			arm3	117	durvalumab	10 mg/kg q2w for up to 12 months	/
			arm4	60	tremelimumab	/	10 mg/kg q4w for 24 weeks then q12w for 24 weeks

CTLA-4, cytotoxic T lymphocyte associate protein-4; PD-1, programmed death 1; PD-L1, programmed death ligand 1; RCT, randomized controlled trial.

### Assessment of risk of bias

A detailed assessment of the risk of bias in the included RCTs is provided in **Supplementary Figures 1**, **2**. The risk of bias for all 10 included randomized controlled trials was systematically assessed using the Cochrane RoB 2.0 tool, revealing a generally high methodological quality. Eight studies exhibited an overall low risk of bias, while two (Cheng Y 2023, Hellmann M D 2019) demonstrated some concerns, predominantly stemming from insufficient reporting details in the randomization process (Domain 1). However, all studies were rated at low risk across other crucial domains, including deviations from intended interventions, missing outcome data, measurement of the outcome, and selection of reported results. Crucially, for the objective primary outcome of overall survival, these potential biases were considered unlikely to fundamentally alter the findings. Therefore, the body of evidence underpinning this meta-analysis is deemed of high quality, supporting the robustness of its conclusions.

### Primary outcome

**Figure 2:**
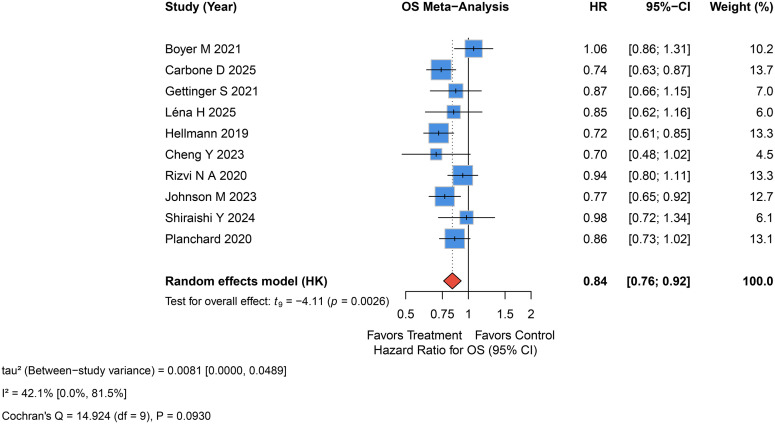
Forest plot of the OS with dual ICI treatment with or without chemotherapy versus control treatment.

All studies reported patients’ OS. The rate of OS in the experiment group was better than control group (HR = 0.84, 95%CI 0.76-0.92, p=0.003, I^2^ = 42.1%, P_Q=0.09, τ²=0.008, 95% PI 0.67, 1.05; **Figure 2**).

Subgroup analysis of OS was performed on sex, age, ECOG status, histological type, region, PD-L1 TPS, TMB, smoking status, immunosuppressant type, brain metastasis, liver metastasis, bone metastasis, regimen of control and treatment line. Subgroup analyses revealed significant OS benefits in the experimental group compared to the control group across the following subgroups: squamous (HR = 0.81, p<0.001), non-squamous (HR = 0.81, p=0.008), PD-1 (HR = 0.84, p=0.016), PD-L1 (HR = 0.84, p=0.002), bone metastases (HR = 0.76, p=0.009), without bone metastases (HR = 0.78, p=0.001). Significant benefits were also observed in male (HR = 0.77, p<0.001), patients aged <65 years (HR = 0.76, p<0.001), those with ECOG = 0 status (HR = 0.76, p=0.038), Asian patients (HR = 0.72, p=0.038), patients with PD-L1 TPS<1% (p<0.001) and PD-L1 TPS ≥50% (p=0.003), those with high TMB (TMB≥10: HR = 0.71, p=0.001; TMB≥20: HR = 0.61, p<0.001), smokers (HR = 0.74, p<0.001), patients with brain metastases (HR = 0.58, p<0.001), those without liver metastases (HR = 0.75, p=0.001), when the control group received chemotherapy (HR = 0.81, p=0.001) or placebo (HR = 0.75, p<0.001), and with those received 1L(HR = 0.83, p=0.001) or non 1L(HR = 0.86, p=0. 046) (**Table 2**).

**Table 2:** Subgroup analysis of the effect of overall survival.

Subgroup	No. of Studies	HR	95%CI	P	Heterogeneity			
					I^2^	P_Q	*τ²*	95% PI
Sex								
Male	6	0.77	0.70, 0.85	<0.001	0%	0.450	0.000	0.68-0.88
Female	6	0.86	0.71, 1.04	0.112	25.9%	0.212	0.014	0.58-1.26
Age								
<65	5	0.76	0.66, 0.88	<0.001	20.7%	0.246	0.005	0.58-1.00
65-74	2	0.85	0.71, 1.02	0.079	0%	0.488	0.000	0.26-2.76
≥75	2	0.93	0.68, 1.27	0.648	0%	0.656	0.000	0.47-1.85
ECOG status								
0	5	0.76	0.59, 0.99	0.038	63.0%	0.035	0.054	0.36-1.61
≥1	6	0.89	0.77, 1.02	0.089	33.0%	0.152	0.009	0.66-1.19
Histological type								
Squamous	7	0.81	0.74, 0.90	<0.001	28.1%	0.292	0.014	0.57-1.17
Non-squamous	7	0.81	0.69, 0.95	0.008	0%	0.455	0.000	0.72-0.92
Region								
Asian	4	0.72	0.53, 0.98	0.038	47.9%	0.139	0.049	0.30-1.72
Not Asian	3	0.81	0.59, 1.13	0.214	83.8%	0.003	0.071	0.21-3.17
PD-L1 TPS								
<1%	8	0.71	0.61, 0.82	<0.001	0%	0.231	0.000	0.61-0.83
1%-49%	4	0.84	0.71, 1.00	0.054	34.6%	0.254	0.013	0.46-1.19
≥50%	7	0.77	0.64, 0.93	0.006	56.2%	0.028	0.029	0.52-1.27
TMB								
TMB≥10	4	0.71	0.58, 0.86	0.001	0%	0.620	0.000	0.51-0.98
TMB<10	4	1.00	0.75, 1.33	0.998	68.3%	0.017	0.050	0.43-2.31
TMB≥20	2	0.61	0.46, 0.80	<0.001	0%	0.953	0.000	0.10-3.56
TMB<20	2	0.98	0.65, 1.46	0.913	88.4%	0.003	0.074	0.01-74.14
Smoking status								
Yes	5	0.74	0.66, 0.82	<0.001	0%	0.404	0.000	0.64-0.86
No	5	1.14	0.91, 1.42	0.249	0%	0.824	0.000	0.83-1.56
Immunosuppressant type								
PD-1	6	0.84	0.74, 0.97	0.016	55.3%	0.051	0.016	0.58-1.22
PD-L1	4	0.84	0.76, 0.94	0.002	19.9%	0.297	0.003	0.67-1.07
Brain metastasis								
Yes	3	0.58	0.44, 0.77	<0.001	3.4%	0.422	0.003	0.30-1.14
No	3	0.88	0.74, 1.05	0.147	58.7%	0.098	0.015	0.46-1.69
Liver metastasis								
Yes	2	0.93	0.73, 1.18	0.536	0%	0.338	0.000	0.20-4.39
No	2	0.75	0.66, 0.85	0.001	0%	0.842	0.000	0.32-1.75
Bone metastasis								
Yes	2	0.76	0.61, 0.93	0.009	0%	0.951	0.000	0.19-2.97
No	2	0.78	0.68, 0.89	0.001	0%	0.576	0.000	0.33-1.86
Treatment line								
1L	8	0.83	0.75, 0.93	0.001	53.2%	0.041	0.013	0.62-1.12
Non 1L	2	0.86	0.75, 1.00	0.046	0%	0.945	0.000	0.34-2.21
Regimen of control								
Monotherapy immunotherapy	5	0.96	0.84, 1.08	0.448	0%	0.619	0.000	0.78-1.16
Chemotherapy	5	0.81	0.72, 0.92	0.001	61.4%	0.222	0.008	0.59-1.11
Placebo	2	0.75	0.67, 0.85	<0.001	0%	0.743	0.000	0.35-1.62

CI, confidence interval; ECOG status, Eastern Cooperative Oncology Group performance status; HR, hazard ratio; PD-1, programmed death 1; PD-L1, programmed death-ligand 1; PI, prediction intervals; TMB, tumor mutation burden; TPS, tumor cell proportion score; 1L, first-line.

In contrast, no significant difference was observed in the following subgroups: female (p=0.112), patients aged ≥65years (65–74 years: p=0.079; ≥75 years: p=0.648), those with ECOG≥1 (p=0.089), non-Asian patients (p=0.214), patients with PD-L1 TPS 1%-49% (p=0.418), those with low TMB (TMB<10: p=0.998; TMB<20: p=0.913), non-smokers (p=0.249), patients without brain metastases (p=0.147), those with liver metastases (p=0.536), and when the control group received monotherapy immunotherapy (p=0.448) (**Table 2**).

Subgroup interaction analyses for OS revealed several statistically significant modulators of treatment effect. A robust interaction was observed with Treatment Regimen Strategy, specifically when comparing Placebo versus Monotherapy immunotherapy (P-interaction<0.0001) and Monotherapy immunotherapy versus Chemotherapy (P-interaction=0.0288), indicating varying relative benefits depending on the comparator arm. Highly significant interactions were also identified for Smoking status (Yes vs No: P-interaction<0.0001), the presence of Brain metastasis (Yes vs No: P-interaction=0.0124), and consistently across TMB subgroups (TMB<10 vs TMB≥10: P-interaction = 0.0330; TMB<20 vs TMB≥20: P-interaction = 0.0192). Conversely, no statistically significant interactions were observed for immunosuppressant type, PD-L1 TPS, ECOG status, age, sex, region, histological type, liver metastasis, bone metastasis, and treatment line, suggesting a generally consistent treatment effect for OS across these characteristics. See **Supplementary Table 2** for details.

### Secondary outcome

**Figure 3:**
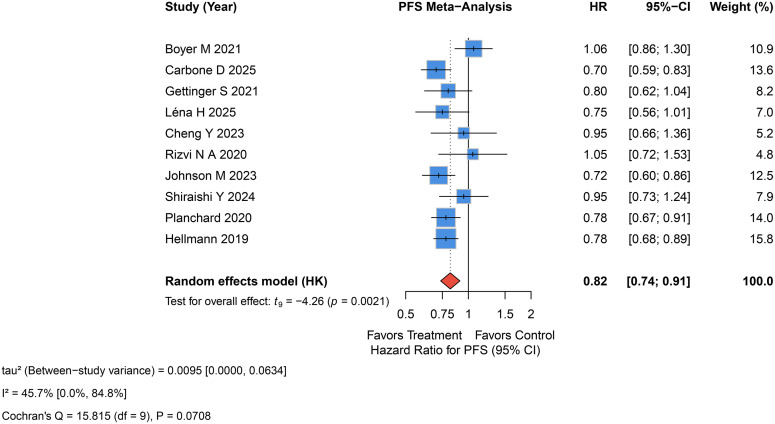
Forest plot of the PFS with dual ICI treatment with or without chemotherapy versus control treatment.

All studies reported patients’ PFS. The rate of PFS in the experiment group was higher than control group (HR = 0.78, 95%CI 0.68-0.89, p=0.002, I^2^ = 45.7%, P_Q=0.07, τ²=0.010, 95% PI 0.64, 1.05; **Figure 3**).

Subgroup analysis of PFS was performed on sex, age, ECOG status, histological type, region, PD-L1 TPS, smoking status, immunosuppressant type, brain metastasis regimen of control and treatment line. The following subgroups showed significant PFS benefits: patients with squamous histology (HR = 0.78, p=0.048), those with PD-L1 TPS<50% (PD-L1 TPS<1%: HR = 0.74, p<0.001; TPS 1%-49%: HR = 0.74, p=0.014), those receiving PD-1 inhibitors (HR = 0.82, p=0.003), those receiving PD-L1 inhibitors (HR = 0.81, p=0.002), patients with brain metastases (HR = 0.54, p=0.034), when the control group received chemotherapy (HR = 0.84, p=0.031), when the control group received placebo (HR = 0.71, p=0.001) and with those received 1L(HR = 0.83, p=0.002) or non 1L(HR = 0.79, p<0. 001) (**Table 3**).

**Table 3:** Subgroup analysis of the effect of progression-free-survival.

Subgroup	No. of Studies	HR	95%CI	P	Heterogeneity			
					I^2^	P_Q	*τ²*	95% PI
Sex								
Male	2	0.82	0.54, 1.24	0.350	86.1%	0.007	0.076	0.01-67.20
Female	2	0.94	0.67, 1.33	0.744	48.9%	0.162	0.030	0.04-21.38
Age								
<65	2	0.83	0.48, 1.45	0.512	88.6%	0.003	0.142	0.00-331.63
65-74	2	0.84	0.69, 1.02	0.079	0%	0.318	0.000	0.24-2.96
≥75	2	1.03	0.60, 1.55	0.888	0%	0.948	0.000	0.07-14.34
ECOG status								
0	3	0.90	0.56, 1.43	0.648	79.9%	0.004	0.124	0.15-5.45
1	3	0.87	0.74, 1.01	0.069	18.7%	0.328	0.004	0.55-1.37
Histological type								
Squamous	4	0.78	0.61, 1.00	0.048	53.1%	0.101	0.034	0.38-1.59
Non-squamous	4	0.87	071, 1.06	0.159	58.5%	0.060	0.023	0.49-1.55
Region								
Asian	3	0.78	0.56, 1.10	0.153	26.7%	0.268	0.026	0.28-2.18
Non-Asian	2	0.89	0.58, 1.38	0.609	89.8%	0.002	0.088	0.01-97.50
PD-L1 TPS								
<1%	6	0.74	0.64, 0.86	<0.001	5.7%	0.380	0.000	0.62-0.89
1%-49%	2	0.74	0.58, 0.94	0.014	0%	0.923	0.000	0.15-3.62
≥50%	4	0.87	0.51, 1.23	0.418	63.8%	0.040	0.066	0.33-2.31
Smoking status								
Yes	2	0.76	0.55, 1.04	0.081	10.7%	0.064	70.8%	0.065
No	2	1.49	1.00, 2.22	0.051	0%	0.664	0%	0.664
Immunosuppressant type								
PD-1	6	0.82	0.72, 0.94	0.003	57.7%	0.043	0.015	0.58-1.18
PD-L1	4	0.81	0.70, 0.93	0.002	9.1%	0.237	0.001	0.63-0.99
Brain metastasis								
Yes	2	0.54	0.31, 0.95	0.034	51.9%	0.150	0.092	0.00-111.54
No	2	0.92	0.66, 1.29	0.636	82.3%	0.018	0.049	0.03-32.15
Treatment line								
1L	8	0.83	0.74, 0.94	0.002	57.8%	0.028	0.016	0.60-1.17
Non 1L	2	0.79	0.69, 0.90	<0.001	0%	0.871	0.000	0.33-1.89
Regimen of control								
Monotherapy immunotherapy	4	0.90	0.78, 1.05	0.179	35.9%	0.210	0.009	0.61-1.33
Chemotherapy	4	0.84	0.72, 0.98	0.031	0%	0.429	0.000	0.65-1.09
Placebo	2	0.71	0.63, 0.80	0.001	0%	0.820	0.000	0.32-1.56

CI, confidence interval; ECOG status, Eastern Cooperative Oncology Group performance status; HR, hazard ratio; PD-1, programmed death 1; PD-L1, programmed death-ligand 1; PI, prediction intervals; TMB, tumor mutation burden; TPS, tumor cell proportion score; 1L, first-line.

In contrast, no significant difference were observed in the following subgroups: male (p=0.350), female (p=0.744), patients with ECOG = 0 (p=0.648), ECOG = 1 (p=0.069), Asian patients (p=0.153), non-Asian patients (p=0.609), patients aged <65 years (HR = 0.83, p=0.512), those aged >65 years (65–74 years: p=0.079; aged ≥75 years: p=0.888), patients with non-squamous histology (p=0.159), those with PD-L1 TPS≥50% (p=0.418), those without brain metastases (p=0.636), when the control group received immunotherapy alone (p=0.179), as well as smokers (HR = 0.76, 95%CI 0.55-1.04, p=0.081) and non-smokers (HR = 1.49, 95%CI 1.00-2.22, p=0.051) (**Table 3**).

For PFS, the interaction analysis identified a more limited set of significant modulating factors. Similar to OS, Treatment Regimen Strategy of the control arm demonstrated significant interactions, specifically for Chemotherapy versus Placebo (P-interaction=0.0310) and Placebo versus Monotherapy immunotherapy (P-interaction=0.0017). Smoking status again presented a highly significant interaction (Yes vs No: P-interaction = 0.0002). Additionally, a significant interaction was found within the Age subgroup, specifically comparing patients aged 65–74 years with those ≥75 years (P-interaction=0.0416). For other factors, including immunosuppressant type, sex, ECOG status, region, histological type, brain metastasis, treatment line, and PD-L1 TPS, no significant interactions were detected, indicating a generally consistent treatment effect for PFS across these subgroups. See **Supplementary Table 2** for details.

### Publication bias and sensitivity analysis

Forest plot of OS and PFS were tested for publication bias and sensitivity analysis in this study. The result of Egger’s test indicates that there was no potential publication bias of OS (**Supplementary Figure 3**, p=0.583). A sensitivity analysis by excluding each of the included studies one by one showed stability (**Supplementary Figure 4**). And the result of Egger’s test indicates that there was no potential publication bias of PFS (**Supplementary Figure 5**, p=0.118). A sensitivity analysis by excluding each of the included studies one by one showed stability (**Supplementary Figure 6**).

## Discussion

Our study found that dual ICI therapy (combined PD-1/PD-L1 and CTLA-4 inhibitors) provides comprehensive benefit for advanced/metastatic NSCLC patients, including improvements in both PFS and OS.

In theory, blocking the CTLA-4 pathway can increase the diversity of T cell receptors, which is particularly beneficial for tumors with a high TMB. High TMB often indicates a higher number of somatic mutations. The somatic mutations that generate new antigens are recognized by the immune system as foreign substances, thereby triggering the activation of cytotoxic T cells and the killing of tumor cells. Therefore, higher somatic mutations can enable the immune system to exert a greater killing effect on tumors when the “braking” mechanism of T cells is relieved by the CTLA-4 inhibitor. Another possible reason is high TMB correlates with increased neoantigen diversity and CD8 T cell infiltration (23), creating an immunologically “hot” microenvironment amenable to checkpoint blockade. Because that the mutational burden determines sensitivity to PD-1 blockade, PD-1/PD-L1 inhibitors combined with CTLA-4 inhibitors can also achieve good therapeutic effects in tumors with a low PD-L1 level (24). Our research results also support this explanation that dual ICI therapy can achieve better therapeutic effects in patients with high TMB. Besides, our research finds patients with low PD-L1 TPS also benefit from dual ICI therapy. In cases where PD-L1 expression is low, somatic mutations may still occur to generate new antigens, which can lead to a higher TMB expression. A study involving 194 patients with advanced NSCLC showed a wide TMB distribution with a maximum of 75 mutations/Mb in a patient population with less than 1% PD-L1 expression (25).

However, the predictive effect of TMB remains controversial. Historically, the predictive value of TMB has been questioned due to the observed disconnect between PFS and OS. For instance, in the CheckMate 227 trial (17), although high TMB (≥10 mut/Mb) predicted PFS benefit, it failed to differentiate OS outcomes, leading to the subsequent withdrawal of the TMB-based indication by Bristol-Myers Squibb. However, in contrast to these findings, our study demonstrates a statistically significant interaction between TMB status and treatment efficacy (TMB = 10: P_interaction=0.033; TMB = 20, P_interaction=0.019), with high-TMB patients exhibiting superior outcomes in both PFS and OS. This discrepancy may stem from variations in TMB cut-off definitions, sequencing platforms, and the inherent heterogeneity of tumor immune microenvironments. Despite the challenges regarding standardization and the limitations of TMB as a standalone marker, our results suggest that TMB remains a potent predictor of response to dual immunotherapy when appropriately stratified. Moving forward, rather than dismissing TMB, clinical efforts should focus on integrating TMB with other immune-related parameters to refine patient selection and optimize therapeutic outcomes.

Beyond TMB, our comprehensive subgroup interaction analyses offer vital insights into the potential heterogeneity of dual ICI therapy efficacy across various patient characteristics. For OS, significant interactions were observed with the Treatment Regimen Strategy of the control arm (indicating varying relative benefits depending on the comparator), smoking status (suggesting differential benefits for smokers vs. non-smokers) and the presence of Brain metastasis (highlighting unique challenges in this subgroup). Conversely, immunosuppressant type, PD-L1 TPS, ECOG status, age, sex, region, histological type, liver metastasis, bone metastasis, and treatment line did not demonstrate significant interactions for OS, suggesting a relatively consistent treatment effect across these factors. For PFS, significant interactions mirrored OS findings for Treatment Regimen Strategy and Smoking status. Additionally, a significant interaction was found within the Age subgroup (65–74 vs. ≥75 years), potentially reflecting age-related differences in immune function or treatment tolerance. Other factors showed no significant interactions for PFS. These findings are crucial for guiding personalized treatment decisions and underscore the need for further validation of identified biomarkers, especially TMB, and continued investigation into the biological underpinnings of differential responses in specific subgroups.

Our meta-analysis indicates that the incremental benefit of adding CTLA-4 inhibitors to PD-1/PD-L1 blockade is limited in patients with high PD-L1 expression and there was no significant difference in the level of PD-L1 according to the interaction p test, likely because therapy with PD-1/PD-L1 inhibitors has demonstrated substantial efficacy in this population—a finding supported by prior meta-analyses reporting a significant overall survival advantage versus chemotherapy (HR 0.31, p<0.001) in patients with PD-L1 TPS≥1% (26). Consequently, the addition of CTLA-4 inhibitors may not confer further survival improvement but can increase the risk of treatment-related adverse events, including higher rates of grade 3–4 toxicities and toxicity-related mortality, as evidenced in comparative studies of monotherapy versus combination regimens (11, 27, 28). Notably, while dual ICI therapy showed a significant OS benefit compared to chemotherapy or placebo, no such advantage was observed when compared directly to PD-1/PD-L1 inhibitor monotherapy. This suggests that the apparent superiority of combination therapy in some comparisons may partly reflect the established efficacy of PD-1/PD-L1 blockade alone, particularly in patients with high PD-L1 expression, for whom monotherapy may represent an optimal balance of efficacy and safety (26).

Based on the above information, TMB may be a more reliable biomarker for predicting the efficacy of dual ICI therapy in advanced/metastatic NSCLC compared to PD-L1 TPS. Studies have shown that TMB is an independent prognostic factor for the efficacy of immunotherapy, unaffected by the expression levels of other indicators (such as PD-L1) (29). Currently, many studies have demonstrated that patients (including NSCLC) with higher TMB in tumors have better responses to PD-1/PD-L1 inhibitor. Our research results also found that patients with high TMB are more likely to benefit from dual ICI treatment. When the TMB was below the threshold of 10 or 20 mutations per megabase, there was no statistically significant improvement in OS and PFS for these patients. Therefore, regardless of whether the threshold is set at 10 or 20, TMB can serve as a biomarker for predicting dual ICI therapy.

This mechanism also explains the most significant finding in this analysis: the significant difference in outcomes between smokers and non-smokers. Smokers experienced a significant improvement in OS, while non-smokers did not benefit even showed a trend towards deterioration. This disparity may be mainly attributed to TMB. Tobacco carcinogens cause extensive DNA damage (30), resulting in a high TMB and a rich neoantigen pool, creating a highly immunogenic TME that is conducive to the action of dual ICI. Combination therapies, especially CTLA-4 blockade therapy, are particularly effective against tumors rich in neoantigens. Conversely, the tumors of non-smokers typically have a lower TMB, providing fewer targets for the reactivated immune system. In this case, the strong but non-specific immune activation triggered by the combination therapy may not effectively target the tumor, and may even increase the adverse reactions of the patients (31), leading to their inability to tolerate the treatment, thus explaining the observed trend towards deterioration.

Beyond TMB, the patient’s intrinsic immune status is another key factor influencing immunotherapy outcomes. Our research has revealed that the patient’s health condition is also of great significance in generating an effective anti-tumor immune response. Significant OS benefit was observed in younger patients (<65 years) and the significance of the interaction p test. This aligns with the understanding that a robust and resilient immune system is a prerequisite for the efficacy and tolerability of intensive immunotherapy regimens. Older patients (≥75 years) may experience immunosenescence and decreased physiological reserve, which can attenuate treatment response and increase the risk of immune-related adverse events, thereby diminishing clinical benefit. Immune aging can lead to problems such as genomic instability, telomere loss, epigenetic disorders, mitochondrial dysfunction and chronic inflammation in immune cells. These factors collectively drive immune cell dysfunction and systemic immunosuppression (32). Moreover, immune aging can also reshape the TME by recruiting immunosuppressive cells (33), senescence-associated secretory phenotypes (SASP) (34), and metabolic reprogramming (35), resulting in treatment resistance and poor prognosis in elderly patients.

Our subgroup analysis based on metastatic sites revealed that patients with brain metastases derived significant benefit from dual immunotherapy. A potential explanation is that brain metastases can disrupt the blood-brain barrier integrity, allowing circulating immune cells to access the brain (36). Another possible reason is that chemotherapy drugs may have difficulty reaching the brain to exert their effects compared to immunotherapies (37). Therefore, patients can obtain great benefits during the double-immunotherapy.

**T**he consistent efficacy of dual PD-1/PD-L1 and CTLA-4 blockade across both first-line (1L) and subsequent-line settings stems from their complementary mechanistic synergy within the cancer immunity cycle (38, 39). In the treatment-naïve 1L setting, CTLA-4 inhibition acts early in the draining lymph nodes to promote *de novo* T-cell priming and broaden the T-cell receptor repertoire, while PD-1 blockade acts downstream in the tumor microenvironment (TME) to restore T-cell effector function and prevent exhaustion (40). Conversely, in later-line settings, prior cytotoxic therapies often induce a highly immunosuppressive TME and resistance to single-agent PD-1 inhibitors (41), yet they simultaneously trigger immunogenic cell death and the release of diverse tumor neoantigens (42). In this context, the addition of a CTLA-4 inhibitor becomes crucial to drive the expansion of a new wave of naïve T-cells against these chemotherapy-induced neoantigens, while concurrent anti-PD-1 therapy protects them from apoptosis. This dual-phase activation effectively overcomes acquired immune resistance, providing a biological rationale for the sustained clinical activity observed even in heavily pretreated tumors (40, 41).

In our subgroup analyses, we observed variations in the pooled effect sizes across different clinical categories, including histology (squamous vs. non-squamous), sex, ECOG performance status and the presence of liver metastases. However, formal tests for interaction revealed no statistically significant heterogeneity (all P > 0.05). This discrepancy between the observed effect size variations and the lack of significant interaction suggests that the apparent differences in clinical benefit are likely not driven by these specific factors. Such findings may be attributed to imbalances in baseline characteristics across subgroups or limited statistical power to detect true interaction effects. Therefore, these results should be interpreted as evidence that the treatment effect is relatively consistent across these clinical strata, rather than indicative of distinct biological interactions. We suggest that these clinical factors should not be considered as primary modifiers of the treatment outcome in this context.

The transformation relationship between PFS and OS also deserves further exploration. In certain subgroups (male, age ≤ 65 years old, Asian, ECOG = 0, PD-L1 TPS≥50%, smokers), although there was a significant benefit in OS, PFS did not show the corresponding benefit. This can be attributed to various factors. Firstly, immunotherapy takes effect relatively slowly and has a relatively long duration (43). Secondly, the PFS is mainly measured through the imaging assessment of the tumor target lesions. This method may not fully reflect the dynamic changes in the therapeutic effect of the dual immunotherapy and may have a lag phenomenon (44). This indicates that OS is still the most reliable indicator for evaluating the true long-term value of an immunotherapy regimen.

We included 10 high-quality RCTs involving a total of 6,369 patients, comprehensively evaluating the efficacy of PD-1/PD-L1 combined with CTLA-4 dual immunotherapy in advanced/metastatic NSCLC. Through preset extensive subgroup analyses, we revealed the heterogeneous efficacy of this therapy in different clinical pathological characteristic populations, especially in subgroups with high TMB, low PD-L1 expression, smoking, and brain metastasis, showing significant survival benefits, providing important evidence for precise immunotherapy. However, this study also has certain limitations. Firstly, the number of included RCTs is limited, restricting the ability to conduct further subgroup analyses, especially in certain rare clinical characteristics (such as specific gene mutations). Secondly, although a random effects model was used and heterogeneity tests were conducted, there is still some clinical and methodological heterogeneity among the studies, such as differences in patient baseline characteristics, treatment details, and follow-up time, which may affect the combined results. Moreover, all the included studies were published English literature, which may have potential publication bias, although the Egger’s test did not show significant bias. Finally, the sample sizes of some subgroup analyses were small, and the results need to be further verified in larger-scale studies.

## Conclusion

Based on high quality RCTs, this meta-analysis confirms that the dual immunotherapy regimen combining PD-1/PD-L1 inhibitors with CTLA-4 inhibitors can significantly improve the prognosis of patients with advanced/metastatic NSCLC. There are significant differences in efficacy among different subgroups, especially in patients with high TMB, low PD-L1 TPS, smoking, Asian, younger age, male, good physical condition, without liver metastasis, with brain metastasis, when the control group received chemotherapy or placebo, 1L and non 1L, where the benefits are more pronounced. However, the additional value of the dual immunotherapy is limited in patients with low TMB, non-smokers, older age, without brain metastasis and when the control group received monotherapy immunotherapy. These findings suggest that TMB may serve as a complementary predictive biomarker for dual immunotherapy; however, its clinical utility should be interpreted with caution and warrants further validation in prospective, large-scale clinical trials. Notably, although subgroup analyses indicated potential differences in treatment efficacy based on histology, sex, ECOG status, PD-L1 expression, and liver metastasis, formal interaction tests failed to reach statistical significance. Consequently, these variations should be interpreted with caution, as they do not provide robust evidence of differential treatment effects across these clinical subgroups.

## Data Availability

The original contributions presented in the study are included in the article/**Supplementary Material**. Further inquiries can be directed to the corresponding author.

## Supplementary material

Supplementary Figure 1 Risk of bias graph for quality assessment of the included RCTs.

Supplementary Figure 2 Risk of bias summary for quality assessment of the included RCTs.

Supplementary Figure 3 Egger’s test of OS for assessing publication bias of included RCTs (p=0.583).

Supplementary Figure 4 Sensitivity analysis of OS for testing the stability of statistical results.

Supplementary Figure 5 Egger’s test of PFS for assessing publication bias of included RCTs (p=0.118).

Supplementary Figure 6 Sensitivity analysis of PFS for testing the stability of statistical results.
