# Supplementary material for: Efficacy of PD-1/PD-L1 plus CTLA-4 inhibitors in advanced/metastatic NSCLC: a meta-analysis based on RCTs
Source: Front Immunol. 2026 May 11;17:1833277. doi: 10.3389/fimmu.2026.1833277 (PMC13199360; doi:10.3389/fimmu.2026.1833277)
Supplement: Supplementary file 1 [file Table1.doc]

**Supplementary Table 1. Characteristics of all the studies included in the** meta-analysis.

| Author | Year | Clinicaltrials.gov Number | Treatment Line | Chemotherapy regimens | TNM stage | Classification version | Performance Status |
| --- | --- | --- | --- | --- | --- | --- | --- |
| Boyer M | 2021 | NCT03302234 | Treatment-naïve (1L) | / | IV | version 8 of the AJCC | ECOG PS≤1 |
| Carbone D P | 2025 | NCT03215706 | Treatment-naïve (1L) | Chemotherapy alone | IV/recurrent | NA | ECOG PS≤1 |
| Gettinger S N | 2021 | NCT02785952 | Previously treated with chemotherapy (2L, ICI-naïve) | / | rcurrent/IV sqCLC | NA | Zubrod performance status≤1 |
| Léna H | 2025 | NCT03351361 | Treatment-naïve (1L) | Standard of care (carboplatin + pemetrexed) | IV/stage III non-treatable by radiotherapy or surgery | NA | PS=2(age<70)/PS≤2(age≥70) |
| Hellmann M D | 2019 | NCT02477826 | Treatment-naïve (1L) | Platinum-doublet chemotherapy | IV/recurrent | NA | ECOG PS≤1 |
| Cheng Y | 2023 | NCT02542293 | Treatment-naïve (1L) | Investigator's choice platinum-based chemotherapy | IV | NA | WHO PS≤1 |
| Rizvi N A | 2020 | NCT02453282 | Treatment-naïve (1L) | Platinum-based chemotherapy | IV | version 7 of the IASLC Staging Manual in Thoracic Oncology | WHO PS≤1 |
| Johnson M L | 2023 | NCT03164616 | Treatment-naïve (1L) | Platinum-based chemotherapy | IV | version 8 of the IASLC Staging Manual in Thoracic Oncology | ECOG PS≤1 |
| Shiraishi Y | 2024 | jRCTs031210013 | Treatment-naïve (1L) | Platinum-based chemotherapy | III not suitable for curative radiotherapy, IV /recurrent | NA | ECOG PS≤1 |
| Planchard D | 2020 | NCT02352948 | Previously treated with≥2 regimens (3L+, ICI-naïve) | Standard of care chemotherapy | IIIB/IV | Version 7 of the IASLC Staging Manual in Thoracic Oncology | WHO PS≤1 |

AJCC, American Joint Committee on Cancer; ECOG, Eastern Cooperative Oncology Group; IASLC, International Association for the Study of Lung Cancer; NA, not available; NCT, national clinical trial; NSCLC, non-small cell lung cancer; PS, performance status; RCT, randomized controlled trial; sqCLC, squamous cell lung cancer; WHO, World Health Organization; 1L, first-line; 2L, second-line; 3L+, third-line or later; ICI, immune checkpoint inhibitor.
