# Supplementary material for: Efficacy of PD-1/PD-L1 plus CTLA-4 inhibitors in advanced/metastatic NSCLC: a meta-analysis based on RCTs
Source: Front Immunol. 2026 May 11;17:1833277. doi: 10.3389/fimmu.2026.1833277 (PMC13199360; doi:10.3389/fimmu.2026.1833277)
Supplement: Supplementary file 2 [file Table2.docx]

**Supplementary Table 2. P interaction result of overall survial and progression-free survival**

| **Subgroup** | **Comparison** | **P interaction** |
| --- | --- | --- |
| **Overall survival** | | |
| Immunosuppressant type | PD-L1 vs PD-1 | 0.977 |
| Treatment Regimen Strategy | Placebo vs Monotherapy immunotherapy | <0.001 |
|  | Placebo vs Chemotherapy | 0.243 |
|  | Monotherapy immunotherapy vs Chemotherapy | 0.029 |
| PD-L1 TPS | <1% vs ≥50% | 0.230 |
|  | 1-49% vs ≥50% | 0.411 |
|  | <1% vs 1-49% | 0.762 |
| ECOG status | 0 vs ≥1 | 0.338 |
| Age | <65 vs ≥65 | 0.391 |
|  | 65-74 vs ≥75 | 0.465 |
| Sex | Male vs Female | 0.326 |
| Region | Asian vs Not Asian | 0.578 |
| Histological type | Squamous vs Non-squamous | 0.970 |
| Smoking status | Yes vs No | <0.001 |
| Brain metastasis | Yes vs No | 0.012 |
| Liver metastasis | Yes vs No | 0.072 |
| Bone metastasis | Yes vs No | 0.450 |
| TMB | TMB<10 vs TMB≥10 | 0.033 |
|  | TMB<20 vs TMB≥20 | 0.019 |
| Treatment line | 1L vs Non 1L | 0.524 |
| **Progression-free survival** | | |
| Immunosuppressant type | PD-1 vs PD-L1 | 0.691 |
| Treatment Regimen Strategy | Chemotherapy vs placebo | 0.031 |
|  | Chemotherapy vs Monotherapy immunotherapy | 0.516 |
|  | placebo vs Monotherapy immunotherapy | 0.002 |
| Age | <65 vs ≥65 | 0.791 |
|  | 65-74 vs ≥75 | 0.042 |
| Sex | Male vs Female | 0.609 |
| ECOG status | 0 vs 1 | 0.902 |
| Region | Asian vs Not Asian | 0.627 |
| Smoking status | Yes vs No | <0.001 |
| Histological type | Squamous vs Non-squamous | 0.523 |
| Brain metastasis | Yes vs No | 0.113 |
| Treatment line | 1L vs Non 1L | 0.325 |
| PD-L1 TPS | <1% vs 1-49% | 0.934 |
|  | <1% vs ≥50% | 0.388 |
|  | 1-49% vs ≥50% | 0.331 |

ECOG status, Eastern Cooperative Oncology Group performance status; PD-1, programmed death 1; PD-L1, programmed death-ligand 1; TMB, tumor mutation burden; TPS, tumor cell proportion score; 1L, first-line.
