# Supplementary figures and images for: Efficacy of PD-1/PD-L1 plus CTLA-4 inhibitors in advanced/metastatic NSCLC: a meta-analysis based on RCTs
Source: Front Immunol. 2026 May 11;17:1833277. doi: 10.3389/fimmu.2026.1833277 (PMC13199360; doi:10.3389/fimmu.2026.1833277)

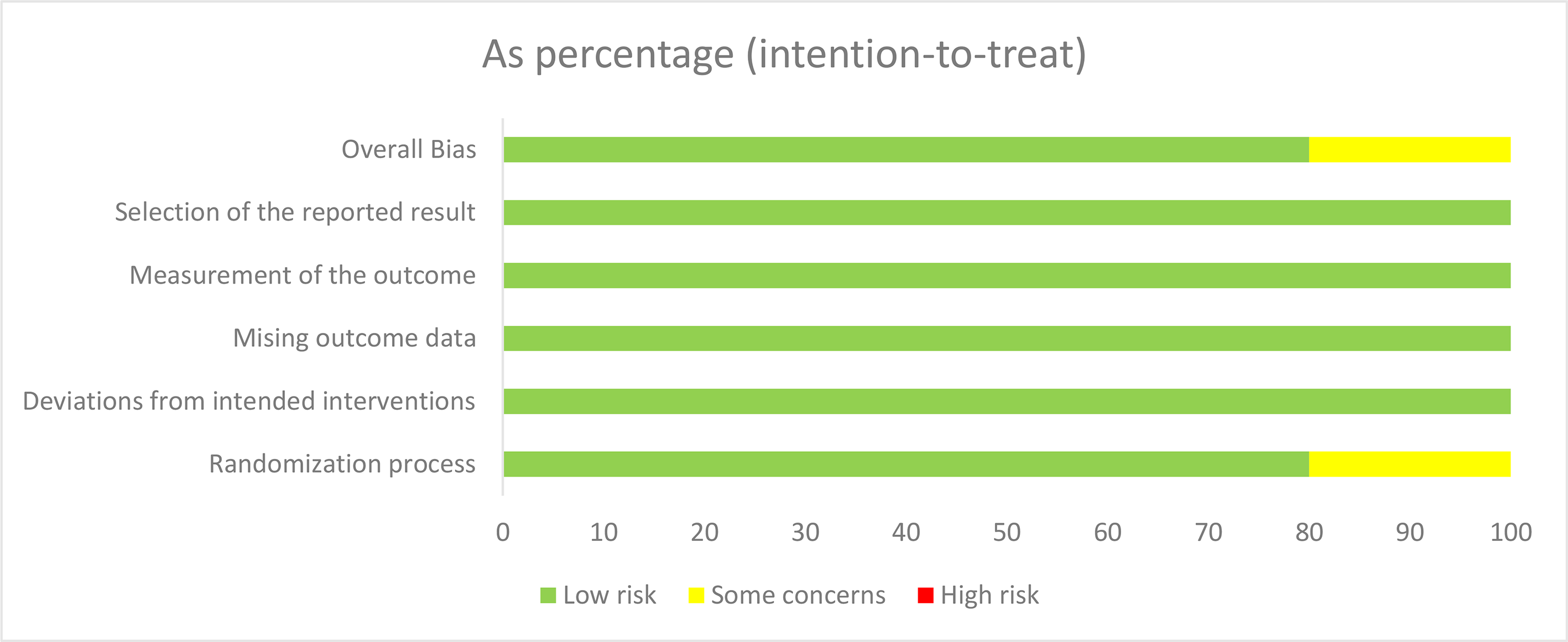

Supplement: Supplementary Figure 1 — Risk of bias graph for quality assessment of the included RCTs. [file Image1.tif]

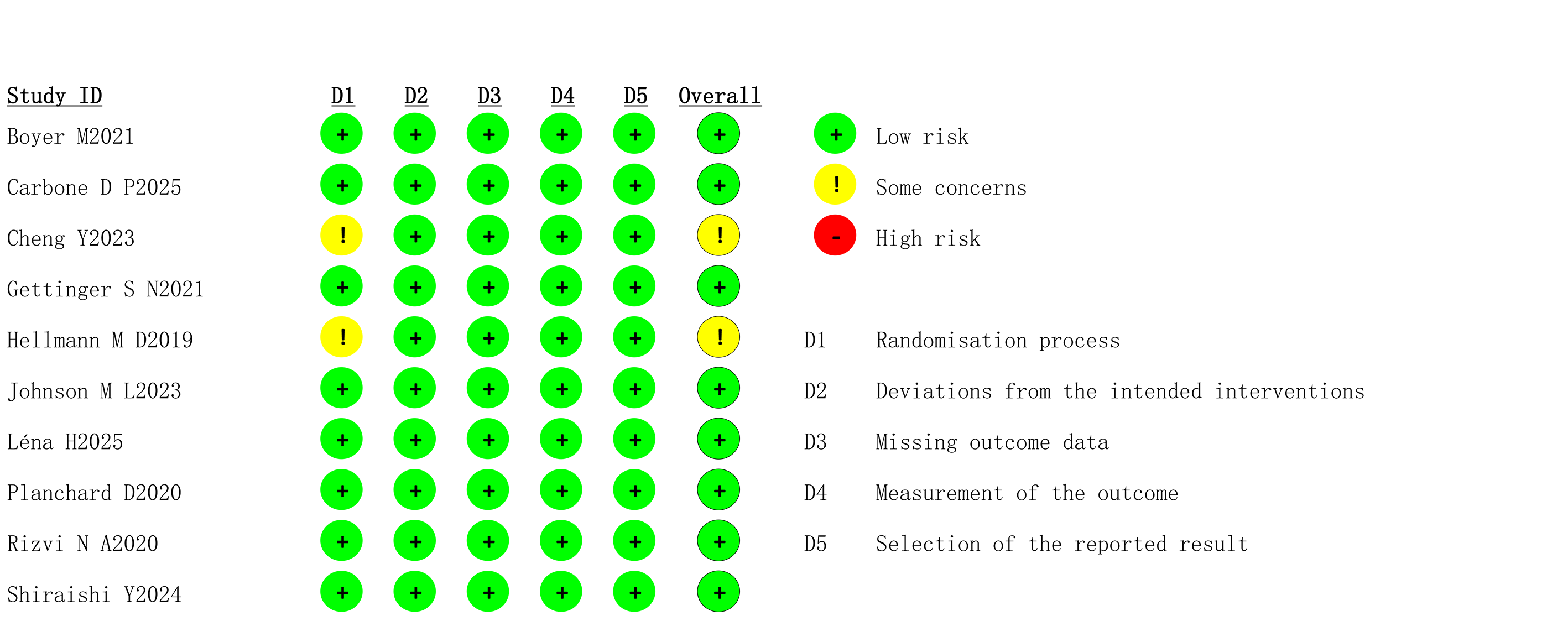

Supplement: Supplementary Figure 2 — Risk of bias summary for quality assessment of the included RCTs. [file Image2.tif]

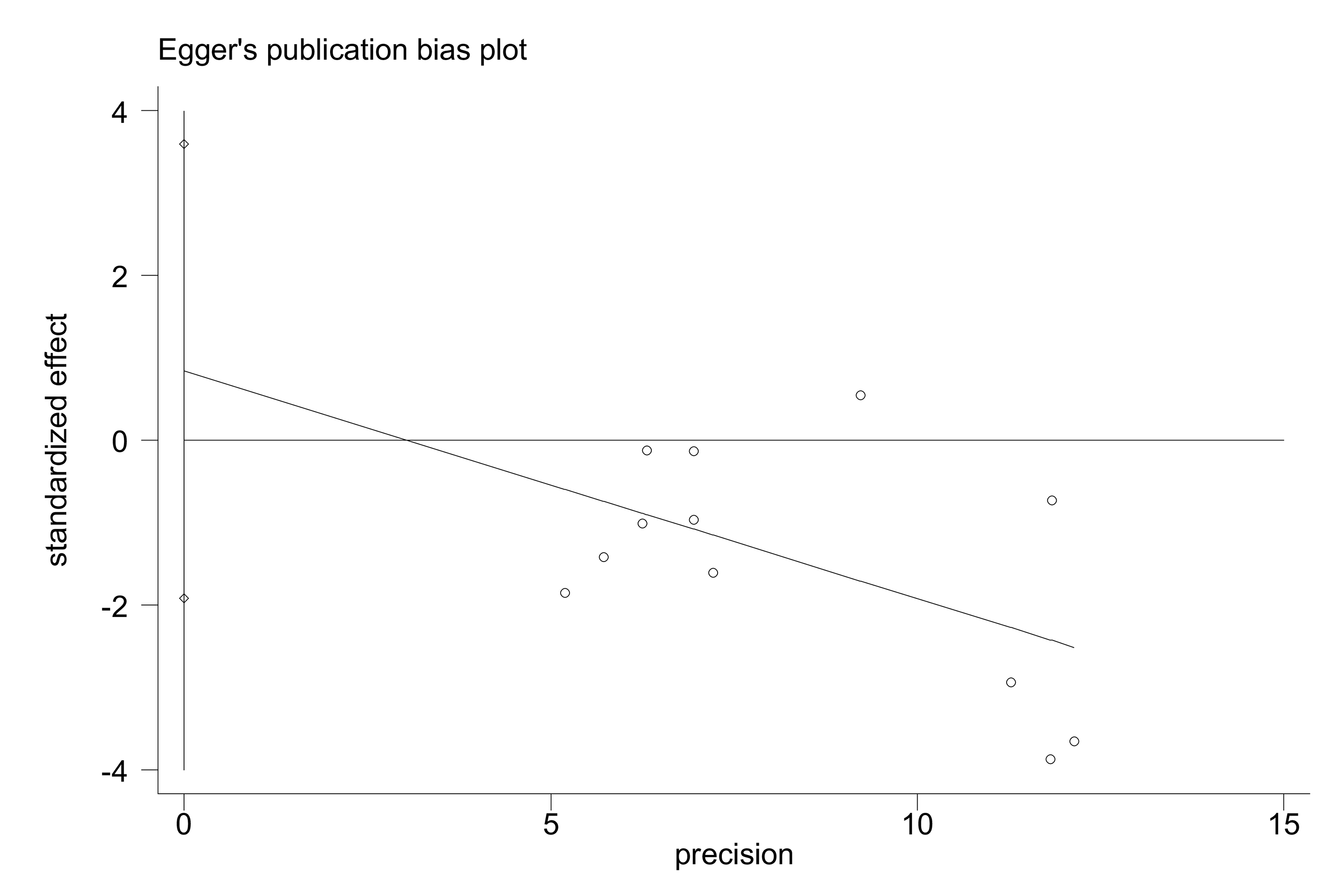

Supplement: Supplementary Figure 3 — Egger’s test of OS for assessing publication bias of included RCTs (p=0.583). [file Image3.tif]

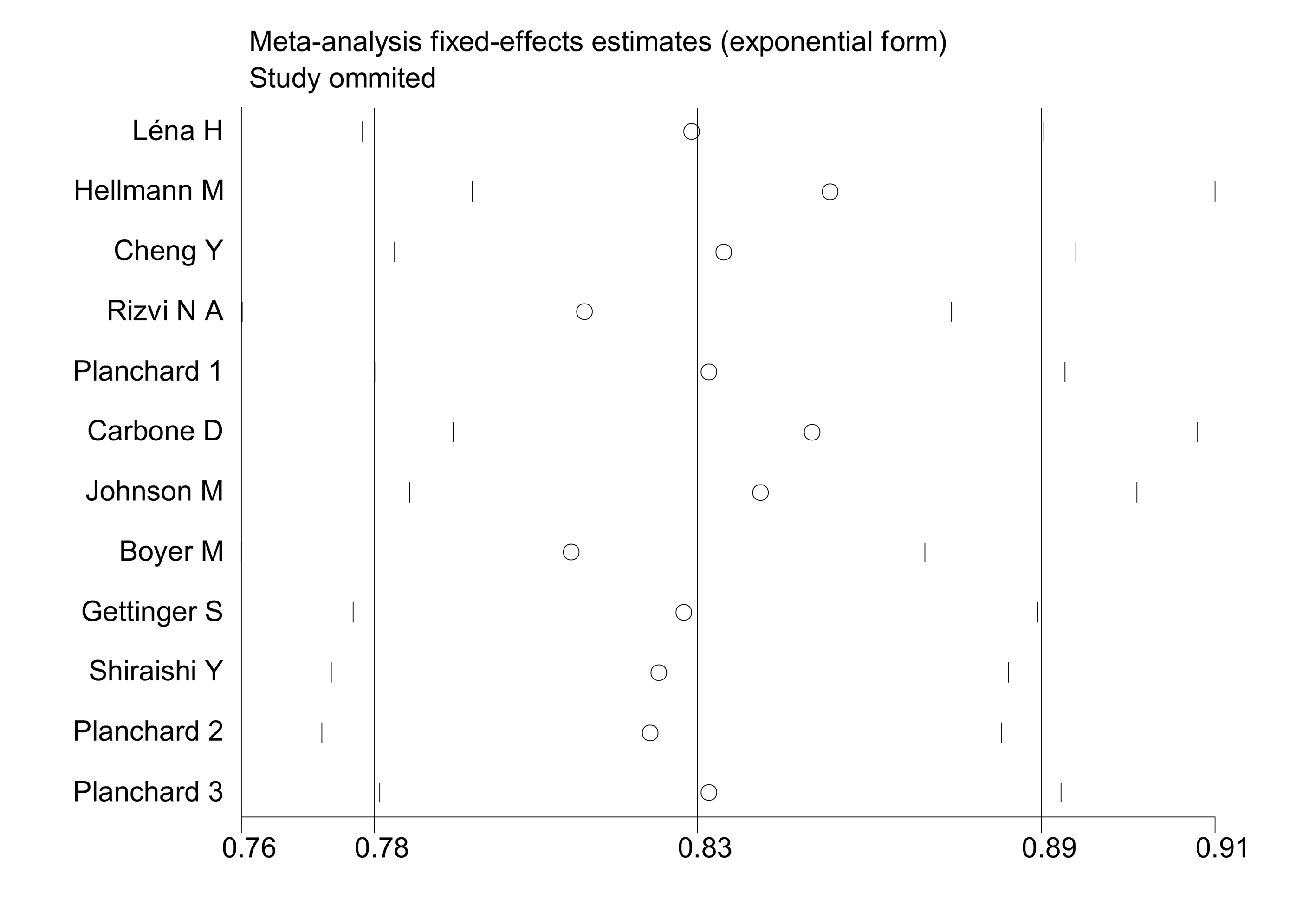

Supplement: Supplementary Figure 4 — Sensitivity analysis of OS for testing the stability of statistical results. [file Image4.tif]

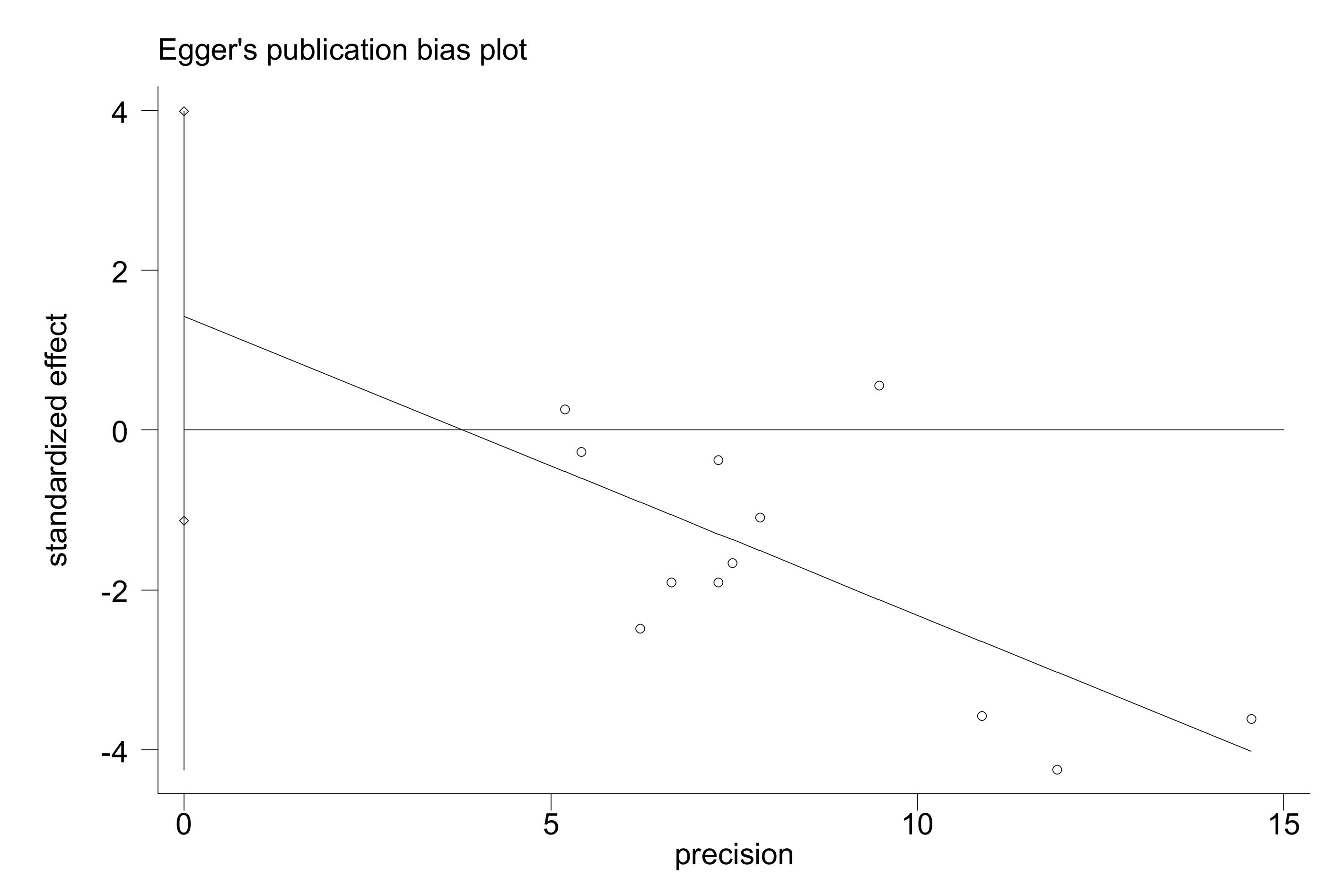

Supplement: Supplementary Figure 5 — Egger’s test of PFS for assessing publication bias of included RCTs (p=0.118). [file Image5.tif]

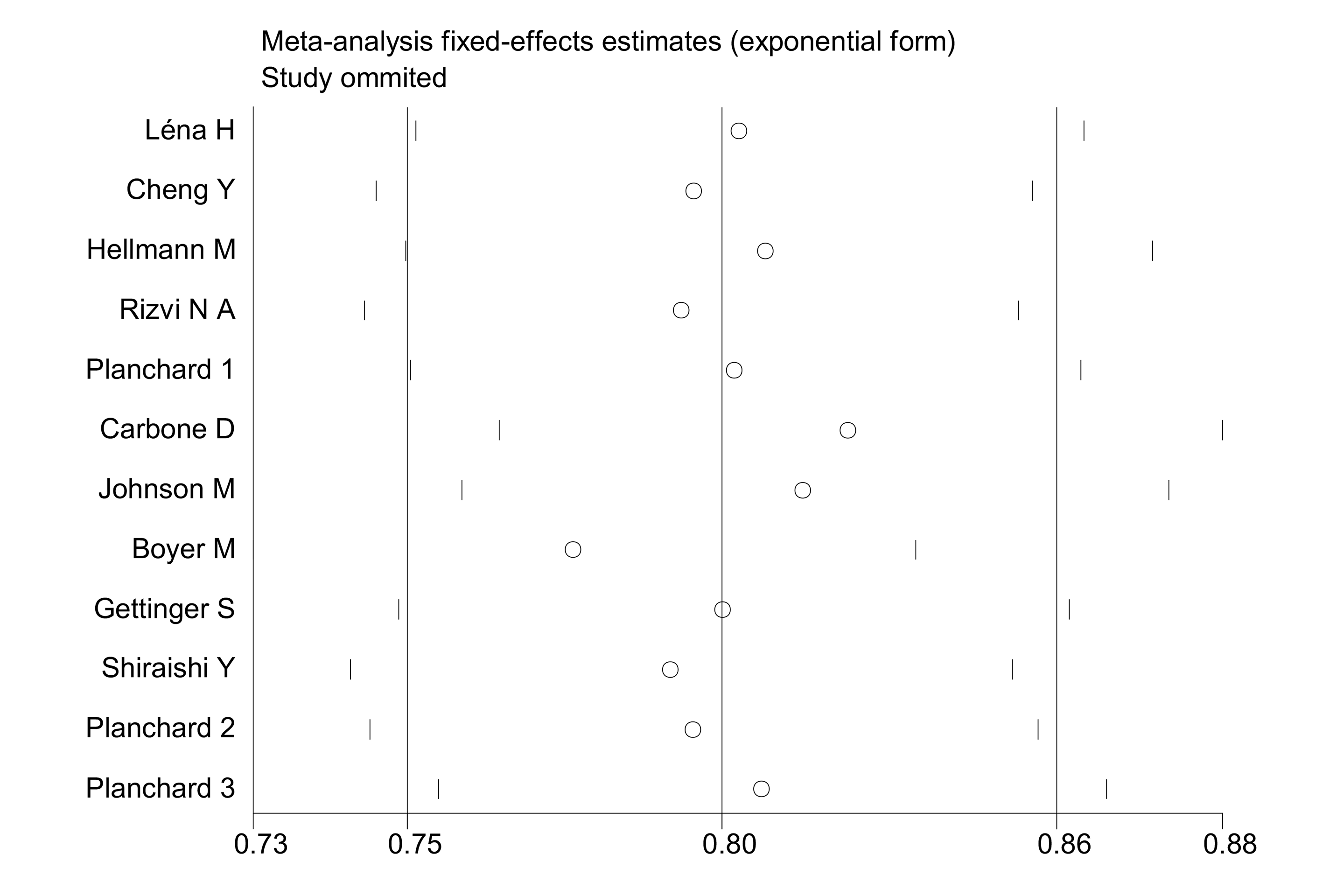

Supplement: Supplementary Figure 6 — Sensitivity analysis of PFS for testing the stability of statistical results. [file Image6.tif]
